# Developing computational biology at meridian 23° E, and a little eastwards

**DOI:** 10.1186/s40709-018-0091-5

**Published:** 2018-11-14

**Authors:** Christos A. Ouzounis

**Affiliations:** Biological Computation & Process Laboratory (BCPL), Chemical Process & Energy Resources Institute (CPERI), Centre for Research & Technology Hellas (CERTH), PO Box 361, 57001 Thessaloníki, Greece

**Keywords:** Computational biology, Bioinformatics, Biotechnology, Biochemical engineering, Science training, Open science, Open research, Science policy

## Abstract

Modern biology is experiencing a deep transformation by the expansion of molecular-level measurements at all scales, using omics technologies. A key element in this transformation is the field of bioinformatics, that has—in the meanwhile—permeated pretty much all of biological and biomedical research and is now emerging as a key inter-disciplinary area that connects the natural sciences, chemical and electrical engineering, science education and science policy, on a number of science and technology fronts. The strong tradition of open access for large volumes of raw data, collections of complex results and high-quality algorithm implementations in bioinformatics makes the field a unique, special case of open science. We report on our recent research activities, the development of training initiatives in the wider region during the past years, and the lessons learned regarding our efforts away from major epicenters, within the general context of open science.

## Introduction

The tremendous progress towards the establishment of computation in virtually every realm of the life sciences and at all scales, with a direct, measurable reference to the molecular level has essentially turned biology into a *bona fide* computational science, with ensuing issues of reproducibility and accuracy as never before [1].

The need for training a new generation of biologists with computational skills and diverse educational backgrounds has also emerged as one of the most important challenges for the life sciences [1]. We report on our recent efforts to develop the field, away from the epicenters of main activity and explore new partnerships in South-East Europe, the Middle East and Africa, and discuss the challenges and opportunities for open science in this general context. In this somewhat autobiographical survey, we point out that one key element connected to open science has always been the availability of a plethora of software tools, data resources and accessible literature including textbooks, that can turn such initiatives into success stories.

## Developing computational biology

In the past 5 years, our efforts have focused on developing the field in new territories—sometimes hindered by economic crisis and other geopolitical factors. In 2014, the Biological Computational & Process Laboratory (BCPL) at the Chemical Process & Energy Resources Institute (CPERI) at CERTH was established and has been in development, with the mission to explore the interface of chemical engineering with synthetic biology and computational biotechnology, as well as advancing the field of computational biology in the wider geographical area.

On the fundamental research front, we are actively working on a number of projects, including phylogenetic profiling [2], metabolic pathway analysis [3], ancestral state reconstructions [4] and radiation exposomics [5]—which are briefly presented below.

We are also actively collaborating with the Universities of Crete and Cyprus, with significant successes in the field of pangenome analysis [6], the inference of functional associations and its validation [7], as well as text mining and concept discovery [8] and software applications such as *BioPAXViz* [9]—driven by open science principles, to ensure both accessibility and reproducibility.

One such collaborative effort with other institutions resulted in the discovery of novel roles for outer ring coat nucleoporins [10] and the detection of a ancestral, universal sequence motif across all known eukaryotic endomembrane coatomers [11].

### Research outcomes

We provide some background about ongoing work as described above, focusing more on the scope of the work and not the details—which can be found in the original publications.

#### Phylogenetic profiling

Phylogenetic profiling is a concise and elegant formalism that allows the summarization of very large amounts of genome-wide similarity studies across a number of species in a tabular format. The x-axis of the two-dimensional array typically corresponds to the number of target organisms while the y-axis is a list of query genes (or protein families): the cells in the table signify presence or absence of similar genes in the target genome. The original formulation consisted of binary values in the similarity matrix [12]; other developments have included real numbers, percentages or weighted values. This elegant framework is widely used in comparative genomics for the genome-wide inference and discovery of functionally linked genes, evolutionary patterns or metabolic networks. As data sizes grow, there is a constant need for faster and accurate methods of phylogenetic profiling. We have devised a new algorithm that uses fuzzy vectors, transformed into discretized binary vectors and a denoising step, used for the estimation of precise distances across profiles. This highly scalable method can then be deployed on a very large scale (millions of genes and thousands of species) to detect consistent similarities, atypical instances and groups of genes for the inference of metabolic pathway presence in entire genomes [2].

#### Metabolic pathway analysis

Thanks to the vast amounts of genomic data obtained over the past 20 years, a wide range of efficient algorithms and well-established methods and workflows, we are now able to process a sequenced genome through a series of computational steps to produce a quantitative metabolic flux model [3]. These steps produce a steady-state model, with constant concentrations and balanced fluxes of reactants and products; these reactions are expressed as a set of constraint equations and submitted to linear optimization in order to maximize biomass production [3]. This computational technique, while still in development, allows massive experiments on a genome scale for the design or modification of organisms for biotechnology and bioengineering.

#### Ancestral state reconstruction

Coupling phylogenetic profiling (essentially summaries of very large comparative genomics analyses) with metabolic pathway modeling (essentially systems-biology type of cell simulations), one can envisage that we can start identifying the building blocks of biochemistry across evolutionary time. Indeed, there have been efforts to reconstruct ancestral genomes all the way to the Last Universal Common Ancestor (LUCA) and estimate their gene content with remarkable consistency [4]. This line of research provides opportunities for synthetic biology designs, where reconstructions (or indeed, likely ‘retrodictions’) of chains of events that led to extant gene distributions can guide experimentation for the synthesis of ancestral genomes or their key sections, e.g. conserved pathways for biotechnological applications (Psomopoulos et al., submitted manuscript).

#### Low-dose ionizing radiation exposomics

In collaboration with a number of key laboratories working on systems radiobiology and genome biology, we are working towards the identification of molecular biomarkers at different levels (genes, proteins, networks) for the detection of effects from low-dose ionizing radiation (LDIR) on the mammalian brain. These processes are just beginning to be understood, and refer to the wider area of exposomics, namely the genome-wide response to environmental factors, in this case LDIR. We have developed a light-weight data integration platform to store previously discovered molecular signatures as well as newly detected gene expression and protein interaction patterns related to the particular study, in anticipation of larger datasets that will be included and made publicly available in the future [5].

All the research outcomes described above are driven by the BCPL at CPERI/CERTH documented in the corresponding publications, listed in the “References” section. We have avoided adding additional references, as these can be found in the original published reports.

### Domain-specific applications

We provide some background about domain-specific applications which represent collaborative efforts with other laboratories, again highlighting the main points—details can be found in the original publications.

#### Pangenome analysis

We have performed the analysis of pangenomes for a group of intracellular parasites with variable genome sizes, the *Chlamydiales* [6]. We have developed workflows for the efficient and accurate detection of protein families, species- and strain-specific genes that can be important for infectious properties, host interactions and functional roles for these groups. The pangenome analysis pipeline relies on a number of previously developed, publicly available tools that have been used extensively in genome comparison studies—such as BLAST [13] and TribeMCL [14]—and is both scalable and generally applicable.

#### Validation studies

High-throughput studies need to be complemented by validated gold-standard datasets. In a particular instance of genome-wide inference of protein interactions, namely gene fusion analysis, there has been an acute lack of clearly defined and experimentally verified predictions. Thus, while computational methods have the potential to be used in high-throughput experimental settings in functional genomics and proteomics, there is a need for high-quality, validated datasets to ensure reproducibility and assess the accuracy of both predictions and experimental results. As a first step, we have discovered a small number of pairwise protein interaction cases derived from small-scale experimental studies that connect the computational inference methods via gene fusion to strong experimental evidence from structural biology, genetics and biochemistry [7]. This catalog has been made publicly available and will be extended in the near future (Tasakis & Ouzounis, unpublished).

#### Genome analysis and annotation

In collaboration with the Universities of Crete and Cyprus, we have developed text mining tools such as BioTextQuest [15] and BioTextQuest(+), enabling cross-database querying, abstract retrieval and entity recognition for optimal document clustering and concept discovery. With multiple analysis options and a Google-like query box, there is much functionality by advanced parameterization available to power users [8].

Significant, long-term collaborations have also been established with the University of Toronto [10] and the Joint Genome Institute at the LBL (Berkeley Lab) [16]—details can be found in the cited references herein. Other recent reports involve the development of HipMCL [17] and perspectives on genome annotation [18].

### Advanced training

Finally, we have invested in teaching and training across our wider region. Key elements in this effort were the series of EMBO practical courses “Bioinformatics and Genome analyses”, with the author as an invited instructor in Athens (2014) and Izmir (2016), and as co-organizer in Thessalonica (2017)—see also http://meetings.embo.org/event/17-genome.

Another major effort during 2015 has been the H3Africa-funded Computational Metagenomics Workshop in Mauritius, supported by H3ABioNet [19]. This significant initiative, at the time of the Ebola virus outbreak, has brought CERTH’s logo and know-how at the southernmost part of the Indian Ocean, in far-flung territories. The overall feedback and the subsequent and continuing collaboration with the University of Mauritius signify the importance of these efforts in areas outside the radar of scientific publishers and science headlines [20]. The gain is of course reciprocal, as we are also learning about regional needs, establish productive collaborations, create added value and exchange students or course materials.

### Lessons learned

This brief, virtually autobiographical report on our efforts during the past 5 years or so, in an area of cutting-edge research and development, should not be seen as a story of a smooth ride. Multiple challenges had to be met, often with minimal resources and adverse socio-economic conditions. None of the research outcomes, applications and training activities reported above would have been possible without a foundation built elsewhere, strong links with overseas laboratories, targeted funding for training actions and, last but not least, unrestricted access to the world’s biological data resources. The lessons learned are valuable both for colleagues in the sciences attempting to conduct research away from the epicenters of their fields, as well as for policymakers who need to (re-)examine certain implicit or explicit assumptions that may not be universally applicable.

First, we have realized that there is a really high demand for research training and scientific exploration everywhere, and at all levels—i.e. undergraduate, graduate, doctoral and post-doctoral training and research [21]. This need is all the more important as the activity is far from the epicenters of a specific field, in this case computational biology and biotechnology. People are willing to travel, of course, to reach the major centres of activity—at the same time, they appreciate immensely a connection with the local context, namely the history, geography, customs and needs of their communities, regions, societies or countries. ‘Localizing’ open science is therefore an element that challenges the notion that all science is global, representing the universal quest for knowledge. The latter notion holds for general scientific principles; yet: local conditions, observations, needs, and specificities also need to be taken into account.

Second, we have discovered that the design, implementation and maintenance of infrastructure elements [e.g. 22] is not always straightforward. These elements, contrary to immediate expectations, are not only computational infrastructures, for example servers or networks, but also the invaluable services of people who maintain these components. Economic, bureaucratic, (anti-)social or regulatory obstacles that can confound the establishment of well-run infrastructures can be the norm rather than the exception, in certain regions—including Greece. Policymakers should be aware that the training of bright young people as aspiring scientists is only one aspect of scientific development of a nation; the aspect of sustainable infrastructure should also be considered as an absolute requirement. Failing that, the danger of brain drain looms large [23].

Third, we experienced the value and importance of major common infrastructure efforts, especially at the periphery. In our area of activity, the European Bioinformatics Institute (EBI) near Cambridge (UK), an EMBL Outstation, plays a key role in collecting, processing and distributing open-access, high-quality data and standards, as well as software services to the global community, beyond the member states and their European partners [24]. Other infrastructures such as the EGI (European Grid Infrastructure), Elixir and GÉANT2, sometimes invisible to the end users and less critical for major hubs, play an equally important role. The key element in this context is the personal experience of having been on both sides of the fence: large, high-‘impact’ institutions and small, lower-‘impact’ laboratories—this dual experience can be both productive and traumatic, yet highly recommended, at some point in one’s career! Note that capable colleagues and competent collaborators can be found in ‘high’ and ‘low’ places. Importantly, when away from the epicenters, it is important to forge partnerships with major institutions and set common goals with partners, sometimes in a difficult, unbalanced process. Asking for help is one thing, forging a common agenda is another: large institutions might appreciate the fact that providing local context and listening carefully to the needs of specific communities can be hugely, and mutually, beneficial.

Fourth, creating and maintaining strong collaborations across institutions of dissimilar size is a real challenge as needs and requirements and, occasionally, the pace can differ. While there are exemplary cases of open science across multiple partners, pairs or groups of institutions, there are many instances where an unbalanced situation requires particular attention—as smaller partners are usually in a disadvantageous negotiating position. Agreements, mutual visits, other forms of trust building, regular updates and common goals or deadlines can remedy these situations with surprisingly positive outcomes. Large institutions, as collaborators and hubs of activity, need to maintain a certain level of reciprocity: this holds at any level, i.e. a ‘small’ institution in Greece compared to their ‘large’ partner institution in the USA can be considered ‘large’ when compared to one in another, smaller, or perhaps less developed country. Size matters, and yet size is relative, therefore policymakers as well as scientists should be aware of this aspect of scientific collaboration—see also below, the last two points.

Fifth, one specific element in the global science enterprise that creates imbalances and usually arises from publishers and their best ‘customers’—i.e. large, high-profile institutions, is the less-discussed aspect of ‘address bias’. Decision makers in, or perhaps in addition to, the scientific community can be oblivious to the vast human potential that remains untapped around the world, and that with little help or advice other regions might benefit and develop scientific research. This ‘subtle process of discrimination’ [25] is amplified by ‘high-impact’ journals which might not consider manuscripts for publication on an equal basis, without reference to the address(es) of the authors—beyond and above the actual content of a study. Unfortunately, we have experienced this situation first-hand and in peculiar ways. Anecdotally, years ago we submitted a manuscript from Greece to a high-impact journal; no need to disclose which one. When I moved to London, we re-submitted the same manuscript (with minor corrections) with the same co-authors and the exception that my address as corresponding author had changed from @[].gr to @[].uk—the manuscript was immediately accepted. This odd example unequivocally demonstrates a situation that many of us may experience. Address bias can be detrimental to science and does skew ranking schemes of academic institutions.

Finally, and given all of the above, let us now consider the true nature of open access, as a small part of open science—other aspects are open research, open data, open source (software). Open research ensures reproducibility [26], open data in all aspects of human activity provides seamless access to information and accelerates research [27] and of course open-source software in all its incarnations facilitates assessment of computer code by users and developers and allows further contributions by entire communities. And yet, funding such efforts on a small scale is a challenge: in particular, open access publications cost—sometimes too much. As journals published by academic societies were lost to commercial publishers (with notable, important exceptions of course), their legacy was relinquished to private companies. Valuable brands and names built over time by incredibly talented scientists driven by unparalleled quality and uncompromising thirst for originality were offered to private enterprises for rather little, I am sure. The end result is a publishing business for science, with profit as one key element. The open access counterculture has tried to address this deficit, by offering another model of publishing: the problem, of course, is the significant cost of each publication—not always taking into account much of the above (human potential, infrastructure availability, ‘localization’ and other issues discussed already). It seems as if in the twenty first century, most science publishing has slowly yet steadily turned into a media world, where publicity and publication lines blur. As a young postdoctoral fellow in the mid-1990s, I was able to publish in subscription-based journals up to a dozen manuscripts a year; today, as a director of research I would need to secure the equivalent of a year’s postdoctoral salary funds to publish the same number of manuscripts as open-access. My productivity has not (hopefully) decreased: my capacity to publish has, as the lack of funding and resources sometimes renders open access publishing an impossible task, further skewing any productivity metric. This element of science policy needs to be taken under serious consideration, as it suffocates scientific creativity and original research—performed by lesser means. The argument that open-access costs are a small fraction of a research project is invalid to much of the world, away from well-funded countries, regions and institutions or laboratories.

The connection to many issues above should be obvious: there is a vast, undertapped human potential, with limited infrastructures, skewed collaboration agendas, experiencing lack of reciprocity (at times) or address bias—and open access might not always serve their goals, because of high *relative* costs. These elements are inter-connected and have a strong impact on the outcomes of research activity and measures of productivity in less privileged or less well-known locations: open access specifically and open science generally work well, when most of the above might be non-issues. The present perspective will hopefully shed some light on the ‘other side’ of scientific research and contribute in a positive way towards future solutions.

## Conclusion

In conclusion, we can state that there is an enormous potential for further development of bioinformatics in the periphery and away from the major epicenters of activity, however requiring local, national, and international infrastructures with coordinated research, services and training activities. Multiple efforts are already in place to address the present deficit. Nonetheless, certain assumptions with regard to open science can still be challenged, namely the notion of global science versus local needs, sustainable and common infrastructure development, balanced partnerships, reciprocal and mutually beneficial agreements, the implicit address bias in the publishing world and finally the costs of disseminating research in an open access mode, for publications (open access), software (open source) or generally scientific results (open science).

## Abbreviation

LDIR: low-dose ionizing radiation
